# We can work it out: an enactive look at cooperation

**DOI:** 10.3389/fpsyg.2014.00874

**Published:** 2014-08-08

**Authors:** Valentina Fantasia, Hanne De Jaegher, Alessandra Fasulo

**Affiliations:** ^1^Centre for Situated Action and Communication, Department of Psychology, University of PortsmouthPortsmouth, UK; ^2^IAS-Research Centre for Life, Mind, and Society, Department of Logic and Philosophy of Science, University of the Basque CountrySan Sebastián, Spain; ^3^Centre for Computational Neuroscience and Robotics, University of SussexBrighton, UK

**Keywords:** cooperation, development, autism, infancy, social interaction, participatory sense-making

## Abstract

The past years have seen an increasing debate on cooperation and its unique human character. Philosophers and psychologists have proposed that cooperative activities are characterized by shared goals to which participants are committed through the ability to understand each other’s intentions. Despite its popularity, some serious issues arise with this approach to cooperation. First, one may challenge the assumption that high-level mental processes are necessary for engaging in acting cooperatively. If they are, then how do agents that do not possess such ability (preverbal children, or children with autism who are often claimed to be mind-blind) engage in cooperative exchanges, as the evidence suggests? Secondly, to define cooperation as the result of two de-contextualized minds reading each other’s intentions may fail to fully acknowledge the complexity of situated, interactional dynamics and the interplay of variables such as the participants’ relational and personal history and experience. In this paper we challenge such accounts of cooperation, calling for an embodied approach that sees cooperation not only as an individual attitude toward the other, but also as a property of interaction processes. Taking an enactive perspective, we argue that cooperation is an intrinsic part of any interaction, and that there can be cooperative interaction before complex communicative abilities are achieved. The issue then is not whether one is able or not to read the other’s intentions, but what it takes to participate in joint action. From this basic account, it should be possible to build up more complex forms of cooperation as needed. Addressing the study of cooperation in these terms may enhance our understanding of human social development, and foster our knowledge of different ways of engaging with others, as in the case of autism.

## INTRODUCTION

The ability to cooperate has received increasing attention over the past years, particularly by researchers from analytical philosophy, and from developmental and comparative psychology. Cooperation has been described as the “coordinated, synchronous activity that is the result of a continued attempt to construct and maintain a shared conception of a problem” (Teasley and Roschelle, 1993) or, more basically, as consisting in “(i) acting or working together and (ii) a common or the same end or purpose” (Tuomela, 2000, p. 3). One of the reasons why cooperation has been considered such an important topic in the past two decades, is its apparent importance in exploring differences between humans and other animals (especially great apes; Tomasello et al., 2005; Tomasello, 2009). Moll and Tomasello (2007, p. 1) have argued that “among primates, humans are by far the most cooperative species, in just about any way this appellation is used (…) constituted by all kinds of cooperative institutions and social practices with shared goals and differentiated roles.”

Despite its extensive exploration by philosophers and psychologists, a clear description and understanding of what makes an activity cooperative is still controversial. This is because cooperation is often described, by mainstream accounts, as depending on high-level social skills, and this, as Butterfill (2012) puts it, “already presupposes too much sophistication in the use of psychological concepts” to be applicable in the investigation of more basic forms of cooperation. Indeed, most of the empirical studies on children’s cooperation are based on inferential and mentalistic theoretical accounts, which may not be the most adequate framework to study how it emerges in typical and atypical developmental paths.

In this paper we challenge these theoretical models and propose to widen the exploration of what cooperation is, what kind of experiences may support someone’s cooperative participation in joint actions, and how this participation may develop over time. Widening the concept of cooperation, we aim to explore the different interactional modalities for it to work out, including those that are not explicitly or previously agreed on as cooperation. We end by drawing some implications of such a change in perspective for cooperation in infancy and in autism.

### PHILOSOPHICAL ACCOUNTS OF COOPERATION

Current theories of joint action have attempted to describe cooperation as a phenomenon primarily based in cognitive abilities. These theories depict social encounters (and cooperative actions) as encounters of minds, where participants have to infer each other’s beliefs and desires to understand and predict the other’s intentions and moves. Central to these theories is the concept of shared intentionality. Many philosophical theories propose that joint actions require the creation of shared (or collective, or joint) intentions^1^ (Gilbert, 1989; Bratman, 1992, 1993; Searle, 1995; Tuomela, 1995). Sharing intentions is possible when partners make individual plans for achieving a common goal, and then formulate predictions upon the other’s intention to achieve the same goal (Gilbert, 1989, 2000; Bratman, 1992; Tuomela, 1993, 2005; Pacherie, 2006). Shared intentions, according to Bratman (1992) are defined as a set of interrelated individual intentional states. In shared activities, he claims, “each agent intends that the group perform the joint action in accordance with and because of meshing subplans of each participating agent’s intention that the group so act.” (Bratman, 1992, p. 333). According to this view, a joint activity is the result of a shared intention, and a shared intention is simply a pattern of “interlocking” plan-intentions of the participants about which they have common knowledge. Essentially, for cognitivist philosophical approaches, partners engage in cooperative actions if they are able to infer each other’s thoughts and plans, and combine them to build their co-actions in some shared way.

As the interest in exploring joint intentionality and joint actions has grown, further theorization followed the original descriptions of cooperation. Building up on Bratman’s account, for example, Tummolini (2013) suggests that representing one’s own goal and those of others from a third-person observational perspective is also a necessary cognitive ability to collaborate, along with mind-reading. Thanks to this allocentric representation of goals (as he names it) individuals are endowed with both “an intention in favor of the joint action and one in favor of a joint mode of reasoning,” which enables them to coordinate in a joint action. Other researchers have attempted to formulate less cognitively demanding accounts of shared intentionality, yet still considering representing intentions at the very ground of any joint action. Sebanz et al. (2006, p. 70), for instance, proposed that a successful joint action “depends on the abilities (i) to share representations, (ii) to predict actions, and (iii) to integrate predicted effects of own and others’ actions.”

Because they presuppose the presence of high-level socio-cognitive capacities, standard accounts of cooperation hardly apply to those who do not possess propositional knowledge about others’ intentions, such as young children or animals, and some philosophers have already questioned this assumption. Tollefsen (2005), for instance, argued that awareness of another’s intention may not depend on inferring it, but on the ability to track intentions-in-action. She argues that attending to each other’s actions provides participants with a shared perceptual space constructed through joint attention dynamics. In this shared space, intentions-in-action are perceptually overt and identifiable so that even young children without a “robust theory of mind” (p. 81) can theoretically engage in cooperative activities. Despite these developmental concerns, the author explicitly renounces to address how this perspective can be effectively applied from very early in development, by saying that “[p]rior to the first year, young infants are like windowless monads” (p. 80), implying that they cannot yet interact. By stressing the importance of joint attention and social referencing mechanisms (as defined by Tomasello, 1995) for the building up of a shared space, she neglects the possibility of earlier forms of cooperation, e.g., in infancy. Similarly concerned with understanding the role of joint action in development, Butterfill (2012) proposed to replace the concept of shared intentions with that of shared goals. Sharing a goal, in his view, only requires agents’ goal-directed actions to be coordinated, but does not imply knowledge. This move should make cooperation possible in early development. However, he also claims that possessing a shared goal requires representing goal-directed actions, and the way this is achieved by young children, in his proposal, is not completely clear.

We find all these arguments to reflect a general problem with the cooperation research reviewed so far: cooperation is framed in its full-blown, adult form and therefore it is difficult to see how those who do not have high socio-cognitive skills (including representing goal-directed actions) or experience could possibly cooperate. This is our main concern in the present paper.

### COGNITIVE DEVELOPMENTAL ACCOUNTS OF COOPERATION

Defining what is to cooperate from a developmental point of view is challenging. Recent developmental research in psychology has endorsed a cognitivist account of shared cooperative activities, suggesting that a major step in children’s social cognitive development occurs when, at around 12–14 months, children begin to engage with adults in cooperative activities involving an understanding of interdependent roles (Tomasello et al., 2005), and are generally motivated to help the other to accomplish her role if needed (Moll and Tomasello, 2007). Therefore, in order to cooperate, it seems that “children must be able to represent, monitor, and regulate both their own and the partner’s behavior relative to their relation to a single, common goal” (Brownell and Carriger, 1990, p. 1165).

To empirically investigate early cooperative skills through abilities such as perspective taking and understanding of the other’s intentions and goals, most of the studies on young children have adopted specifically designed lab tasks involving role reversal or simultaneous coordination of movements (Brownell and Carriger, 1990; Warneken et al., 2006, 2012). In the majority of these studies, successfully performed joint tasks would set the age threshold for attributing cooperative abilities and instrumental helping to children.

For example, Brownell et al. (2006) observed children at 19, 23, and 27 months of age engaging in peer cooperative problem solving tasks. In these tasks, each child had to pull simultaneously or sequentially one handle of a wooden box to activate a musical toy mounted on the box. Activating the toy by coordinating each other’s timing and movements would lead to successful performance of the task. The researchers found that 1-year-old children coordinated their actions more by coincidence than in a cooperative way, whereas older children appeared to be more actively cooperating toward a shared goal. They took these results to confirm their view that the ability to cooperate depends on “being able to represent and to share goals and intentions with a partner” (p. 806); an ability that, according to the study, could only be seen over the second and third years of life. Another example is a study in which Warneken and Tomasello (2007) investigated instrumental helping and cooperation in 14-months-olds children. Instrumental helping was defined as providing help to people in completing a task, e.g., pick up an out-of-reach object, whereas cooperation was measured through a series of cooperative tasks to be resolved jointly, such as retrieving an object from a vertically movable cylinder embedded in a platform. Results showed that at 14 months children reliably helped a partner who could not achieve a goal, but cooperated successfully only in tasks demanding low coordination. The authors concluded that “Helping might be easier for children than cooperating because it requires the understanding of what another individual intends to do (…), whereas cooperation requires the ability to form a shared goal and to mesh plans of action toward that goal” (ibid. p. 291). In other words, helping would only require to read another’s intention, whereas cooperation would also need for one’s own and the other’s intentions to be co-dependent and converge.

In sum, developmental research has attempted to define the beginning of cooperation by setting tasks based on similar premises, thus designing practical tasks that need not only inferring but also mobilizing well-formed intentions to be completed. These premises derive from the mainstream philosophical accounts of cooperative actions, which propose that to be engaged in a cooperative action requires possessing mind-reading abilities, and abilities to align one’s own intentions and beliefs with the other’s, although milder, less cognitively weighted positions have also been proposed. In the next section we will discuss what we believe are some pitfalls of both the existing theoretical and methodological approaches to the study of cooperation.

### METHODOLOGICAL AND THEORETICAL ISSUES WITH STANDARD APPROACHES

To put shared intentionality at the very basis of shared cooperative action raises the question of how humans get to know others’ intentions and goals. On the standard accounts, this is done by use of a theory of mind or a simulation mechanism, which is “any cognitive system … that predicts or explains the behavior of another agent by postulating that unobservable inner states particular to the cognitive perspective of that agent causally modulate that agent’s behavior” (Penn and Povinelli, 2008, p. 394). This cognitive system is often thought to be supported by the so-called social brain (Frith and Frith, 2003; Frith, 2007).

#### If intentions are hidden, are joint intentions hidden too?

Within mind-reading approaches, social understanding requires, among other things, being able to get access to another’s intentions, or more in general, contents of the mind. The “problem” of understanding others’ minds is based on the premise that intentions are hidden and private, that is, that others’ intentions (like thoughts, ideas, beliefs) need to be inferred through complex representational operations (Apperly, 2011). Now, how are such intentions shared? On standard representationalist accounts, this is often proposed to happen through some forms of mental alignment, for instance by simultaneous mirror system activation (Gallese, 2003; Pacherie, 2006; Sebanz et al., 2006). In this view, everyone has her own understanding of others’ intentions to jointly perform an action, but how these understandings become shared remains unclear. For example, Knoblich and Sebanz (2008) have attempted to explain how people can form intentions to act together in three steps. First, they need to be able to derive the other person’s intentions behind her object-directed actions or actions directed to her partner. Then, actors need to be able to keep knowledge of these intentions separate from their own intentions. Eventually, “There needs to be an intentional structure that allows an actor to relate his/her own intention and the other’s intention to an intention that drives the joint activity” (Knoblich and Sebanz, 2008, p. 2025). Although it may seem very basic, this definition is still quite cognitively demanding, and does not solve the main problem of how an “intentional structure” works. Is it individual or shared, implicitly or explicitly created?

There seems to be a gap here in the form of an empty space *in between* people: these approaches have explained shared intentionality from an observer’s perspective, but not from a participant’s one. This is in line with criticisms of the standard approach to social cognition (e.g., Gallagher, 2001; Leudar and Costall, 2009) and with views on interpersonal alignment as primarily based on embodied engagement (Macmurray, 1991; Braten, 2003; De Jaegher and Di Paolo, 2007; Fuchs and De Jaegher, 2009; Reddy and Morris, 2009). Shotter (1983, p. 39) nicely summarized these alternative positions: “Motives, intentions, sentiments are (. . .) directly perceived by those directly involved in [a joint action] as first person actors and second person recipients in that activity. Only third person observers have to make inferences.”

Another consideration is whether we need *to know* that we are cooperating in order to be able to cooperate. Often, cooperation is presupposed as something we set out to do, so that actions are either clearly cooperative or not – a separate and identificable type of action altogether. This may indeed sometimes be the case, for example when two people meet to perform a certain shared task, like bathing a very agitated dog. But taking this idea as the starting point for understanding cooperation presupposes that we already know what it is, and so we do not need to define the elements out of which it could arise. It precludes, for example, the possibility that cooperation arises without there being a predefined intention or motive to cooperate, while this may be key to understanding how people get to cooperate in the first place. Shared goals may emerge during the course of an interaction, and so participants can “roll into” cooperation without having previous awareness of it. For instance, making space for someone who enters a crowded bus is achieved by the new and old passengers together, each adjusting movements and postures. Here, a common goal emerges out of the interaction and in the context of a small space to be shared as smoothly as possible. Understanding this emergent kind of phenomenon will give us further insights into what cooperation is and how it works.

#### Where is development?

We may question to what extent we can explain the role of cooperative actions in children’s development if we conceive of cooperation as heavily relying on high cognitive skills, and a long experience with social interactions. As Butterfill (2012, p. 24) wrote:

If the leading account were the whole truth about joint action, engaging in joint action would presuppose, and therefore not explain, much of the development of reasoning about others’ mental states. (. . .) We need a further account of joint action, one that is compatible with the premise that joint action plays a role in explaining how humans develop abilities to think about minds.

Furthermore, developmental research on cooperation is based on a rather restricted pool of tasks, which are designed to assess cooperative problem solving and related abilities like role reversal, perspective-taking and joint attention. These do not necessarily cover the whole range of possible cooperative interactions in a child’s life, as there are many situations (some of which we discuss below) in which a clear, explicit division of roles and statement of goals is not needed. Furthermore, the structure of these tasks implies a “pass or fail” evaluation and seems therefore more appropriate to detect when cooperative skills are already present, rather than telling us how they emerge or develop in time (Thelen and Smith, 1994).

Which view on cooperation one adopts is likely to have rather serious consequences when studying cooperative exchanges in typical and atypical development. This is, for example, the case with research on cooperation in autism. Studies on cooperation in autism that are based on mind-reading and perspective-taking abilities^2^ find that children with autism are less successful than children with developmental delay (Sally and Hill, 2006; Liebal et al., 2007). However, this does not mean that they are completely incapable. For instance they seem able to help an adult as needed (Liebal et al., 2007), particularly when they understand the other person’s goals toward an object (Aldridge et al., 2000; Carpenter et al., 2001). Liebal et al. (2007) explained these findings in terms of a specific impaired understanding of the partner’s role within the cooperative task that would not apply when the situation does not require knowledge of and agreement on each partner’s role. Thus, it may be that children with autism can succeed in cooperative tasks, if they do not entail an explicit understanding and prior agreement on each partner’s role. Similarly, if they are given appropriate interactive support, e.g., if they are helped with being aware of the other person in the interaction, they can cooperate in a dual-control technology task (Holt and Yuill, 2014).

In conclusion, to study cooperation as it develops and in conditions implying impairments in social skills we need to investigate it at a more basic level than has been done so far. In the next section, we put forward our proposal, which looks at cooperative interactions from the point of view of what is at stake for the individuals participating in them, and the organization of cooperative interaction processes. For doing this, we will use the concepts and research tools of *enaction*, a specific approach to cognition within the embodiment movement in cognitive science (Varela et al., 1991; Thompson, 2007; Di Paolo et al., 2010).

## THE ENACTIVE PERSPECTIVE ON SENSE-MAKING AND SOCIAL INTERACTIONS

Enaction is a non-reductive naturalistic approach that proposes a deep continuity between living and cognitive processes. It is a scientific program that explores several phases along this life-mind continuum, based on six mutually supporting, operational concepts: *autonomy*, *sense-making*, *embodiment*, *emergence*, *experience*, and *participatory sense-making* (Varela et al., 1991; Thompson, 2005, 2007; De Jaegher and Di Paolo, 2007; Di Paolo et al., 2010). Here, we first introduce two of its main concepts: *sense-making*—the enactive notion of cognition in general; and *participatory sense-making*—enactive social cognition. In section 3, we start applying these ideas to understanding cooperation.

### SENSE-MAKING

For enaction, “the mind is seen not as inhering in the individual, but as emerging, existing dynamically in the relationship between organisms and their surroundings (including other agents)” (McGann et al., 2013). Or, as Merleau-Ponty (1962, p. 430) already put it:

The world is inseparable from the subject, but from a subject which is nothing but a project of the world, and the subject is inseparable from the world, but from a world which the subject itself projects.

In this view, the paradigmatic cases of cognizers are living organisms (Varela, 1997; Thompson, 2007). One of their crucial properties is their constitutive and interactive *autonomy*, which is defined as a network of dynamical processes (metabolic, immune, neural, sensorimotor, etc.) that actively generates and sustains an identity under precarious conditions (Di Paolo, 2005). An autonomous system constantly produces itself physically, and regulates its interactions with the world to satisfy the needs created by its precarious condition (Di Paolo, 2005). The living organism spontaneously generates its own goals and responds to the environment (McGann, 2007), in accordance with its self-organization. The cognizer is therefore always situated in a world that is significant for it, based on this perspective based on need. Its world is not pre-given but largely *enacted*, i.e., shaped as part of its autonomous activity. For the enactive approach, cognition is *embodied*, meaning that a cognizer’s activity depends non-trivially on the body. The body is more than just anatomical or physiological structures and sensorimotor strategies; it *is* the precarious combination of various interrelated self-sustaining identities (organic, cognitive, social), each interacting with the world in terms of the consequences for its own viability (Di Paolo, 2005).

These ideas together ground the enactive characterization of cognition as *sense-making*: a cognizer’s adaptive regulation of its states and interactions with the world, with respect to the implications for the continuation of its own autonomous identity. The concept of sense-making describes the relation between an autonomous agent and the world of significance it enacts. It therefore does not conceive of cognitive processes as representational and avoids the known problems of cognitivism. Organisms do not passively receive information from their environments, which they then translate into internal representations whose significant value is to be added later. Natural cognitive systems participate in sense-making as a relational and affect-laden process grounded in biological organization (Jonas, 1966; Varela, 1991, 1997; Weber and Varela, 2002; Di Paolo, 2005; Thompson, 2007). Sense-making, thus, is *valued* or *concerned* acting and interacting, leaving no gap between affect and cognition—they are one in the relation of significance between cognizer and world.

### PARTICIPATORY SENSE-MAKING

Having briefly explained what enactive cognition is, and sense-makers’ inherently meaningful perspective on and interactions with the world, let us now take a closer look at social encounters, the second main element in our enactive sketch of cooperation. The enactive approach considers sociality in its broadest form, namely as *intersubjectivity*, or the meaningful engagement between subjects (Reddy, 2008), in which three aspects are crucial: engagement, meaning, and subject. Meaning and subjectivity have been explained above in terms of sense-making, namely as the way living (cognizing) systems always meaningfully engage with their environment, because they are self-organizing and self-maintaining. In this section, we turn our gaze on engagement between such concerned subjects.

Crucial to the enactive approach is the focus on *social interaction processes*, which are complex phenomena involving different dimensions of verbal and non-verbal behavior, varying contexts, numbers of participants and technological mediation. They impose strict timing demands, involve reciprocal activity, exhibit a mixture of discrete and continuous events at different timescales, and are often robust against external disruptions. Essential to interaction is that it involves *engagement* between agents. Engagement (Reddy, 2008; Reddy and Morris, 2009) captures the qualitative aspect of social interactions once they start to “take over” and acquire a momentum of their own. It also reflects the way this experience is described in everyday language (e.g., “being in sync with someone”). Experientially, engagement is the fluctuating feelings of connectedness with one another, including that of being in the flow of an interaction.

In order to capture this taking-over aspect of engagement, enaction defines social interaction in terms of the autonomy (as defined above) of the interaction process and that of the individuals involved, as:

a co-regulated coupling between at least two autonomous agents, where: (i) the co-regulation and the coupling mutually affect each other, constituting an autonomous self-sustaining organization in the domain of relational dynamics and (ii) the autonomy of the agents involved is not destroyed (although its scope can be augmented or reduced; De Jaegher et al., 2010, pp. 442–443; also De Jaegher and Di Paolo, 2007, p. 493).

Apart from each agent involved in such a coupling contributing to its co-regulation, the interaction process itself also self-organizes and self-maintains. To illustrate this, think of how sometimes, when you encounter someone coming from the other direction in a narrow corridor, you end up in front of each other, then each step aside, moving to the same side at the same time, preventing both of you from continuing on your way. This simple example shows how the interaction process can become autonomous or “take on a life of its own.” At the same time, the interactors also maintain their autonomy as participants. This is a necessary condition for calling an interaction social, because if one of the participants loses their autonomy, for the other it would be like interacting with an object or a tool, and thus not a social interaction anymore (De Jaegher and Di Paolo, 2007).

Social interactions are sustained by processes of embodied coordination, including its breakdowns and repairs (De Jaegher and Di Paolo, 2007; Di Paolo and De Jaegher, 2012). Coordination does not necessarily require cognitively complicated skill. Analyses of social interactions and conversations in social science show that participants can unconsciously coordinate their movements and utterances, and this is already the case in mother-infant interactions (Condon and Sander, 1974; Stern, 1977/2002; Condon, 1979; Scollon, 1981; Davis, 1982; Tronick and Cohn, 1989; Kendon, 1990; Grammer et al., 1998; Malloch, 2000; Jaffe et al., 2001; Issartel et al., 2007; Malloch and Trevarthen, 2009). With the concept of coordination and other dynamical systems tools, interaction dynamics can be measured (see e.g., Kelso, 2009). Moreover, they can be related to neural activity (see e.g., Lindenberger et al., 2009; Dumas et al., 2010, 2012; Cui et al., 2012; Di Paolo and De Jaegher, 2012; Konvalinka and Roepstorff, 2012; Schilbach et al., 2013).

Based on this definition of social interaction, and the notions of sense-making and coordination, we can now characterize social understanding as *participatory sense-making*: If, as indicated above, we make sense of the world by moving around in and with it, and we coordinate our movements with others when interacting with them, this means that we can coordinate our sense-making activities. That is, we literally *participate in each other’s sense-making activities*. Thus, on the enactive account, social understanding is understood as the generation and transformation of meaning together in interaction (De Jaegher and Di Paolo, 2007; De Jaegher, 2009; Fuchs and De Jaegher, 2009). Participants co-create the interactive situation, *but also* the interaction process as such influences the sense-making that takes place. If a social interaction is as characterized, then people can act together, also for no apparent end or purpose of their own, or even against their individual ends (e.g., the corridor encounter). Even without a shared intention to start with or when entered into against their will by the participants, interacting can change or affect one’s ends or purposes.

This has an interesting consequence for understanding intentions, namely they are truly generated and transformed interactionally, and interacting with each other opens up new domains of sense-making that we would not have on our own. This contrasts with the way intentions are conceived in cognitivist approaches to cooperation, as introduced above, namely as hidden, and only shareable by high-level cognitive mechanisms. On our account, intentions do not first arise or are first made individually, but they emerge as the interaction goes on (Di Paolo, under review). Therefore, intentions are visible and understandable by each participant, also in cooperative interactions, as they are contextualized and stem from that specific ongoing interaction. This makes understanding and aligning with the other’s intentions un-mysterious: it happens in doing things together, which is moving together, since movements are already and always imbued with meaning for sense-makers (Johnson, 2007; Sheets-Johnstone, 2011; Merritt, 2013). On the basis of this, we can see how intentions can evolve in their jointness, meanings and specificity for those involved throughout interaction, including cooperative ones.

## COOPERATION AS A PROCESS

Here, we start from the most rudimentary or minimal form of cooperation, in order to make it understandable from a developmental point of view. With the enactive concepts of sense-making and participatory sense-making in hand, let us now look again at cooperation, starting from its basic definition as “(i) acting or working together and (ii) a common or the same end or purpose” (Tuomela, 2000, p. 3). Now, considering social interactions as already cooperative in a basic sense (in line with our enactive approach), we want to characterize our approach to cooperation starting from this definition by Hubley and Trevarthen (1979, p. 58):

cooperation means that each of the subjects is taking account of the other’s interests and objectives in some relation to the extrapersonal context, and is acting to complement the other’s response.”

In our view, “taking account of the other’s interests and objectives” does not need inferencing, as we argued, but happens through embodied interactions that are meaningful in the given situation and in the interactional history. These actions are complementary in that they fit each other in some form. This is not only the case for positive co-operations but also for situations in which we argue and disagree about something, where some complementarity is still needed in order for the disagreement even to be played out. This means that there are different forms, layers, and aspects of cooperation: embodied, in time, in space, in topic, imitative or complementary, etc. The fact that we are interacting guarantees that some basic cooperative layer is present (e.g., in the corridor situation, we cooperate to stop cooperating) and therefore, every time we interact, we cooperate, in a basic sense. Also, since sense-making always involves affect, this view of cooperation becomes less intellectualistic and begins to investigate how affective processes may be involved in cooperation. Then, the challenge is to investigate what further levels of cooperation are present in a specific interaction or situation, over and above the basic interaction process. This can involve different, increasingly more complex levels of sense-making.

Like the enactive approach, interactionist approaches such as ethnomethodology and conversation analysis have also based their empirical program on a theory of social interaction as a dynamical constructions and a view of others’ intentions as mutually accessible and accountable for. Ethnomethodology was originally developed by Garfinkel to “discover the methods that persons use in their everyday life (. . .) in constructing social reality” (Psathas, 1968, p. 509), and thus study how this reality is constructed, produced and organized in social encounters. Derived from phenomenology, it shares with it an interest in exploring the participants’ embodied experience of being engaged in mundane interactions; the latter are seen as phenomena in their own right, yet situated in specific cultural contexts and practices (see, for instance, the work of Schütz, 1967/1932). Inspired by ethnomethodology and by ((s))Goffman’s (1983) work on the interaction order, Conversation analysis (Sacks et al., 1974; Sacks, 1992; Schegloff, 2007) investigates the systematic features of naturally occurring conversations. In a large body of work now spanning over five decades, it has revealed the fine, moment-by-moment coordination of speakers, and the sequential structuring that enable the orderly participation of different interactors across turns-at-talk and within complex activities. Central in this approach is a view of human communication as multimodal, where different but integrated communicative resources (verbal and non-verbal) contribute to establishing the interactional context, anticipating, co-constructing, and if necessary repairing the emergent definition of what is going on (Kendon, 1990; Streeck et al., 2011; Tulbert and Goodwin, 2011). Thus, interactions are always cooperative, inasmuch as participants orient to, monitor and support the interlocutors’ understanding and act so as to enable their successive moves (Goodwin, 1995, 2013).

Intentions and goals are not searched before or behind the communicative action as its “cause,” but manifest in speakers’ behavior, shaped and adjusted as the interaction unfolds. Within this framework, and in convergence with enactivism, cooperating is possible even for those – like young children – who do not possess a robust capacity to “read” others’ intentions or plans, but can nevertheless participate in joint, situated interactions (Forrester, 2008; Lerner et al., 2011; Mehus, 2011).

### COOPERATION IN INFANCY

We can now ask what this view implies for understanding cooperation in infancy. Since infants cannot remain alive alone, they need others to help them with nourishing, shelter, hygiene, and social interaction. On our account, it is to be expected that infants contribute actively to this caring, because they are themselves sense-makers, generating and maintaining their own living identity, and also, quite possibly, already their social identity (Stern, 1985/2000; Delafield-Butt and Gangopadhyay, 2013).

Hubley (1983) defined cooperation in infancy as the joint management of objects, actions or ideas to fulfill a purpose that two interactors share. She identified some minimum requirements for cooperative actions in infancy, which are (1) a shared plan of action within mutual orientation, with the infant attending to and acting with reference to the partner’s indicated purposes; (2) active contributions to a single coordinated event, which, on the infant’s part, is seen as a clearly identifiable and oriented action to influence the behavior of the partner and then mesh with the partner’s action to complete a shared purpose; (3) willing participation. On the one hand, such a definition seems fitting with the infant’s limited communicative resources as it does not imply that the partners should verbally agree on a shared plan or goal. However, it presupposes that some shared plan has been somehow established, and requires that each partner understands the interest or purposes of the other regarding the shared action. As we already argued, such an explicit agreement may not be required in all forms of cooperative interactions.

The fundamental contribution of past developmental research has been to reveal how early communicative interactions are created out of contributions of both the infant and the caregiver (Hubley and Trevarthen, 1979; Trevarthen, 1979). Bruner (1977) recognized shared reference and role-taking as cooperative features in communicative interactions involving giving and receiving objects before 1 year of age. More recent observations have demonstrated how, since very early in life, infants adjust and facilitate actions directed to them, especially in daily routines such as when the caregivers pick them up, change their nappy, or play a social game with them (Service, 1984; Nomikou and Rohlfing, 2011; Reddy et al., 2013; Rączaszek-Leonardi et al., 2013; Fantasia et al., 2014). Under a perspective that considers social interactions as basic forms of cooperation by participating in shared, meaningful interactions, infants practice their ability to make sense of and coordinate with the caregiver’s action, becoming increasingly skilled in their social participation.

One of the criticisms we made of existing studies was that they measured children’s cooperative ability when they successfully performed a joint pre-fixed task; regarding cooperation as a cognitive skill that can be switched on and off means neglecting the importance of learning processes that sprout *from* and *within* cooperative interactions. In contrast, in a here and now perspective the process of cooperating enables children to build up their actions moment by moment through a sequence of relational adjustments and (dis-)engagements toward a joint goal. Thanks to its structuring and structured nature, cooperation may be seen as a framework in which development occurs and at the same time as a mode of being with others learnt during development. If we take seriously what was proposed so far – that any interaction requires some basic cooperation, followed, in some cases, by a process of co-negotiation toward a more or less explicit goal that matters to those who are involved in that process – then we may also explain how it develops. And, at the same time, we may be able to see how participating in goal-directed joint actions supports and shapes infants’ development.

### COOPERATION IN AUTISM

A different theoretical perspective may also open up new possibilities for investigating cooperation in autism. As reported above, empirical findings suggest that some children with autism (at different chronological ages) perform poorly in high-level cooperative tasks and in other correlated abilities, such as joint attention, imitation, perspective taking, and role-reversal (see Colombi et al., 2009). Yet, performing “poorly” does not mean that the capacity is absent, and indeed some children with autistic spectrum condition (ASC) do pass the cooperative tasks. This result is not consistent with the theoretical premises informing the design of the tests and the difficulties of children with autism, classically understood. One way to explain this (controversial) evidence may lie in changing the premises we start from, instead of *post hoc* adjustments to the interpretation.

Studies of the verbal production of children with autism that do not start from a deficit but try to understand the children’s spontaneous interactional behavior, can help to illustrate and support this shift of perspective. Conversation analysis studies, for example, allow to observe how even echolalic productions (the repetition of utterances with no apparent relation to prior talk from other speakers), often seen in children with autism, are in fact responsive moves (Loca and Wootton, 1995; Wootton, 1999; Stribling et al., 2005/2006; Sterponi and Shankey, 2014). The repetition of available utterances helps children to stay in the conversation despite their difficulty with improvising a newly designed turn. Sometimes these stereotypical contributions can take the form of questions and feed the progression of an interaction, supporting the child’s continued participation in a social exchange (Sterponi and Fasulo, 2010).

Dickerson et al. (2007) also show that observing what children actually do reveals capacities for cooperation that cannot emerge in pre-defined tasks, for sometimes the ways in which children find solutions for their difficulties are not incorporated into the tasks. They investigated classroom interactions between two autistic children and their tutors. The children were asked to answer questions, using answer-cards. During the session, each of the children tapped the answer-cards, an action which at first sight seemed meaningless. However, using conversation analysis, Dickerson et al. (2007) could show that the children tapped on the cards just before they started answering, and sometimes continuing into their answering. This seems to indicate that the tapping is a way of engaging and of “projecting a relevant forthcoming response on the part of the child” (Dickerson et al., 2007, p. 297). In other words, the children found means to signal their ongoing engagement when the timing of their verbal production was delayed, thus cooperating to the maintenance of the interactive plane.

Using fine-grained observational methods, the actions of all participants can be studied and analyzed in interaction, making it possible to pick up the forms of cooperation that infants and people with autism are capable of (see also Stribling et al., 2009). These examples demonstrate how the use of non-verbal and non-vocal resources for building up a co-participatory model of how the child and teachers work together becomes possible thanks to transcripts of the interactions. In this way, not only the participants’ talk, but also a number of non-verbal activities that are salient for the interaction are acknowledged. These results fit well with the Vygotskian idea that collaborative work leads to learning (Vygotsky, 1978; see also Goodwin, 2013). Furthermore, these studies suggest that ways to observe cooperative interactions in autism exist, if only we consider interactions and autism from a different perspective. During everyday interactions at home or school, in the car or at the park, children with autism are involved in many simpler, not-always-explicit cooperative exchanges. Not only are the children part of these exchanges, but they also grow into them; namely, they learn to be active partners out of everyday cooperative interaction, just like every other child does. This is not to say that there are no difficulties or differences, but social understanding in autism may be more fruitfully studied from thebasic and positive perspective we put forward here.

## IMPLICATIONS

In summary, the perspective shift we propose has implications for understanding development as well as autism. Firstly, our approach supports a developmental stance on cooperation in that it explores how we become cooperative interaction partners in the first place. If we assume high-order mental skills (or a great deal of “social experience”) to be prerequisite for cooperating, we would not able to see how infants can grow into social interactions and gradually learn to engage with the social world around them, but rather wait until much of the development has already happened. If, on the other hand, we propose that cooperation is a form of interacting and understanding each other, it does become possible to investigate how cooperation can emerge and be learnt even in early interactions. In this perspective, cooperation in infancy is a product of development, as well as a process in which development occurs.

An interesting aspect to consider regarding development is how to conceive of cooperation in asymmetrical interactions. Infants seem to be able to cooperatively coordinate with caregivers since very early (see e.g., Reddy et al., 2013; Fantasia et al., 2014), but they may not do it with peers until later on, as suggested by some research (Warneken and Tomasello, 2006, 2007). From an enactive point of view, it is not surprising that infants are better able to cooperate with a caregiver than with a peer, since the presence of someone with more interactive experience makes the overall interaction more competent. This is related to ((s))Vygotsky’s (1978) notion of the zone of proximal development, where it is possible to scaffold someone in interaction to be jointly more capable of activities they cannot yet do alone. What is needed for an interaction to be cooperative if the relation is asymmetric? If we think of a pick up situation, we know that the adult is doing the major part by actually holding the infant and lifting her up. Yet, infants are not passively waiting for it to happen. They make specific preparatory body adjustments that facilitate the mother’s movements, and thus, the pick up sequence (Service, 1984; Reddy et al., 2013). At the same time, when the adult fails to complete the expected pick up sequence, infants seem to stop being cooperative by dropping their body tension and participation (Fantasia et al., 2014). In this case, although the mother has the main role in making the pick up sequence effective, the infant’s role is essential in its being clearly oriented toward the joint achievement of the interaction. Obviously, asymmetry may or may not play a strong role depending on the task.

As a second point, if we are to understand autism in general, and specifically people with autism’s capacity to cooperate (which is firstly a particular form of social interaction) the change of perspective we propose here may also be helpful. We may try to forsake a typical-development perspective and, as Petra Björne and other authors have already suggested, reverse our glasses, paying more attention to what people with ASC can do and the way they describe their own experiences (Björne, 2007; Robledo et al., 2012; De Jaegher, 2013; Donnellan et al., 2013). As shown by the studies on autism presented in the previous section, if we consider actions in their interactional context and in their significance for all participants, it becomes possible to understand the emergence of cooperation also in the interactions of and with people with autism. Exploring cooperation in children with autism from an observer or third-person perspective not only fails to take into account the child’s experience of cooperating as an engaged partner; it also cuts out how the other person is feeling or experiencing the child as a partner. In cases like autism, in which social interactions run a different course, in which jointly attending to an object may not be at the core of the interaction, approaching cooperation from a second person perspective can make all the difference.

We thus suggest that future studies on cooperation and autism should include more ecological observations and parental reports. We expect to gain more detailed knowledge about what infants and children with autism can do cooperatively in early goal-directed interactions from taking an enactive approach. This involves: finely studying the interaction (e.g., through ethnomethodology or conversation analysis), taking into account the context or the environment (using, for instance, parental reports or ecological observations), and studying what is at stake for the individuals involved (i.e., asking how they make sense in and of the interaction).

## CONCLUSION

We hope to have shown that it is possible to encompass a wider range of cooperative interactions, not only those in which interactors explicitly agree upon and set rules and roles for a specific shared task to be performed. This is not to neglect that in some particular scenarios participants do need to make efforts to make sense of the other’s intentions, and indeed goals need to be set out and agreed beforehand. Only, this is not always the case, as cooperation is a multi-layered process that may take different forms. In this perspective, we share Tollefsen’s view that intentions-in-action can emerge out of ongoing interaction (Tollefsen and Dale, 2012), with the minimum requirement that interactors share an interactional space. Cooperation is a form of participating in each other’s sense-making, in which we may form a goal or purpose together while interacting. It is not a skill that can be lacked but rather a way of being with others that is possible to learn. Learning to cooperate then becomes understandable as an important aspect of typical and atypical development. For this reason, we think that future developmental research on cooperation (and social cognition in general) could benefit from more ecological observational methods and less adult-centric approaches (Donaldson, 1978). As the adult’s way of cooperating is an already fully blossomed one, one in which the picture is complete (and intentions can be easily inferred if needed), we need instead to observe infants and their daily living and discover the basic, emerging ways in which cooperation develops.

## Conflict of Interest Statement

The authors declare that the research was conducted in the absence of any commercial or financial relationships that could be construed as a potential conflict of interest.

## Footnotes

1 We will not go into the debate here about specific differences between shared or collective intentionality or other denominations as it is not relevant for our argument. For an overview of analytic standpoints on the terms, see (Schweikard and Schmid, 2013).

2 Mainstream accounts of autism have long proposed that people with autism have difficulties in mind-reading (Baron-Cohen, 1989; Dinishak and Akhtar, 2013), joint attention (Loveland and Landry, 1986), or impairments in turn-taking skills (McEvoy et al., 1993), although these findings are not uncontroversial, and even primary proponents recognize that there is always a number of participants who do pass the tests (Happé, 1994; but see also Boucher, 1989, 1996, 2012; Gernsbacher et al., 2008).
